# Arterialisation of the Portal Vein With an Aortoportal Jump Graft for Portal Vein Thrombosis Following Liver Resection for Malignancy

**DOI:** 10.1155/1999/28282

**Published:** 1999-07

**Authors:** Long R. Jiao, Nagy Habib

**Affiliations:** 1 Liver Surgery Section Imperial College School of Medicine Hammersmith Hospital London W12 ONN United Kingdom; 2 Head of liver Surgery Section Division of Surgery Anaesthetics and Intensive Care Imperial College School of Medicine Hammersmith Hospital, Du Cane Road London W12 0NN United Kingdom

## Abstract

Fibrolamellar hepatocellular carcinoma (FHCC) is a variant of hepatocellular carcinoma, which mainly affects a young age group and carries a relatively good prognosis. It is widely accepted that aggressive curative resection is still the best option for FHCC. We report here a case of successful arterialisation of the portal vein with an aortoportal jump graft for portal vein thrombosis, which developed postoperatively in an already comprised portal vein with tumour invasion following an extensive liver resection for FHCC.


*HPB Surgery*, 1999, Vol. 11, pp. 279–281
Reprints available directly from the publisher
Photocopying permitted by license only

© 1999 OPA (Overseas Publishers Association) N.V.
Published by license under
the Harwood Academic Publishers imprint,
part of The Gordon and Breach Publishing Group.
Printed in Malaysia.

*Case Report*

### Arterialisation of the Portal Vein with an Aortoportal Jump Graft for Portal Vein Thrombosis Following Liver Resection for Malignancy

LONG R. JIAO and NAGY HABIB*

*Liver Surgery Section, Imperial College School of Medicine, Hammersmith Hospital, London W12 0NN*

*(Received 28 April 1998)*

**Fibrolamellar hepatocellular carcinoma (FHCC) is a variant of hepatocellular carcinoma, which mainly affects a young age group and carries a relatively good prognosis. It is widely accepted that aggressive curative resection is still the best option for FHCC. We report here a case of successful arterialisation of the portal vein with an aortoportal jump graft for portal vein thrombosis, which developed postoperatively in an already comprised portal vein with tumour invasion following an extensive liver resection for FHCC.**

*Keywords:* Fibrolamellar hepatocellular carcinoma, liver resection, aortoportal jump graft

### INTRODUCTION

Acute portal vein thrombosis can develop following abdominal surgery or neoplasm [1]. Although controversy exists with regard to treatment of this condition, it is widely believed that aggressive treatment should be administered quickly as this serious complication carries a high mortality and morbidity [2]. Current options for management of acute portal vein thrombosis and transjugular intrahepatic portosystemic shunt, thrombectomy and surgical reconstruction [3–6]. We report here a case of acute portal vein thrombosis in a patient following an extensive liver resection for FHCC and its successful management.

### CASE REPORT

An 18-year-old girl with obstructive jaundice was referred to us for further investigation and management, having been diagnosed as suffering from FHCC. Initial examination was unremarkable except a 3 cm hepatomegaly below the right costal margin.

Imaging studies included an abdominal CT, visceral angiography and percutaneous transhepatic cholangiography. These investigations demonstrated as follows: 1) a space occupying le-

*Corresponding author. Head of liver Surgery Section, Division of Surgery, Anaesthetics and Intensive Care Imperial College School of Medicine Hammersmith Hospital, Du Cane Road, London W12 0NN Tel.: 0181 383 8574, Fax: 0181 383 3212, e-mail: nhabib@rpms.ac.uk sion in the left lobe of the liver invading the medial sector of the right liver involving segments V and VII; 2) encasement of the left branch of the portal vein, middle and left hepatic veins by the tumour, together with a 90% obstruction of the right branch of the portal vein (Fig. 1); 3) presence of hilar lymphadenopathy; 4) obstruction of the left hepatic duct with compression of the right and common hepatic ducts.

In view of the nature of malignancy and her age, she underwent a left hepatectomy extended to segments V and VIII with preservation of the integrity of the right hepatic ductal system and the right hepatic artery. At operation, the portal vein was also explored and a Forgarty catheter was used to remove the invading tumour within the right branch of the portal vein. Hilar lymph node dissection was also carried out and a frozen section confirmed the presence of extrahepatic disease.

Postoperatively, she recovered well initially. However, 3 days later she developed acute thrombosis of the remaining right branch of portal vein which was confirmed on Doppler ultrasound. Faced with this difficult and serious postoperative complication, we decided to perform an urgent thrombectomy to unblock the portal vein. Unfortunately, rethrombosis occurred soon after thrombectomy. Therefore, we proceeded with resection of the thrombosed portal vein and complete reconstruction of the portal stumps. An end to side anastomosis was created between the mesenteric portal stump and the inferior vena cava by using an 8F PTFE ring graft. At the same time, the hepatic stump of the portal vein was anastomosed to the infrarenal aorta with an 8F PTFE ring prosthesis to form an aortoportal jump graft to achieve arterialisation of the hepatic portal vein (Fig. 2). Although postoperatively she developed a transient episode of ascites immediately after surgery that was managed conservatively, she made a full recovery and was discharged home on the 18th postoperative day. She enjoyed a good quality of life for 40 months but died subsequently from tumour recurrence.

### DISCUSSION

Curative resection if the best option for hepatic tumours either primary or secondary [7, 8]. FHCC represents a different entity of hepatocellular carcinoma, carrying a good prognosis. It mainly affects a young age group and at presentation patients usually have evidence of extrahepatic involvement of malignancy [9]. It is widely accep-

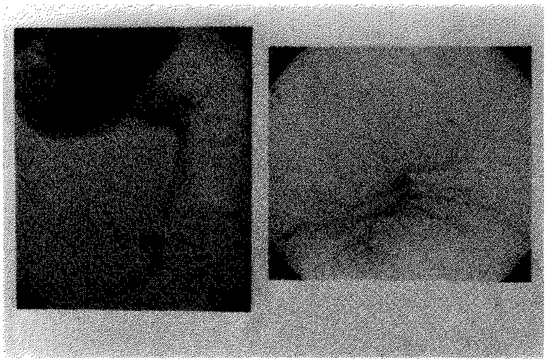

FIGURE 1 Preoperative visceral angiography showing a complete obliteration of the left branch of the portal vein and a near 90% obliteration of the right branch of the portal vein.

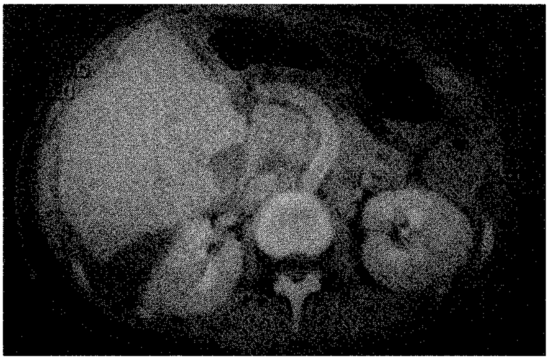

FIGURE 2 Postoperative CT scan showing the aortoportal jump graft.

ted that treatment for this group of tumours should be aggressive surgical resection and where deemed necessary liver transplantation can be performed following a total liver resection [10]. In our case, the patient presented with extraheptic metastases with hilar lymphadenopathy, but with a resectable liver lesion.

The major cause of acute portal vein thrombosis in adults is malignant disease, mainly from pancreatic and hepatocellular carcinoma, constituting 5% to 6% of portal vein thrombosis in the West [2]. Acute portal vein thrombosis can also occur following blunt trauma or intra-abdominal surgery [1]. Without prompt treatment, it is invariably fatal in the acute situation especially when complicated by small bowel infarction. In our patient, the acute portal vein thrombosis developed as a result of a combination of direct invasion by the tumour and surgery within the portal vein.

Arterialisation of the hepatic portal vein with portocaval shunt has been performed successfully in patients with portal vein thrombosis undergoing liver transplantation [12]. Temporary arterialisation of the portal vein was also described during liver transplantation [13, 14] and liver resection [15]. Here, we report the first of such cases following liver resection for neoplasm. We feel that although it is technically challenging, arotoportal jump grafting could be achieved as a salvage procedure for patients with portal vein thrombosis following liver surgery who would otherwise have an extremely grave prognosis. We also postulate that this technique might be useful in extreme cases of liver resection in order to achieve maximal tumour clearance.

### *References*

[1] Tscholl, D. J. (1980). Venous thrombosis in the portal venous system and liver cirrhosis. Paraneoplastic syndrome?, *Arch Anat Cytol. Pathol.*, **28**, 163–168.

[2] Witte, C. L., Brewer, M. L., Witte, M. H. and Pond, G. B. (1985). Protean manifestations of pylethrombosis, A review of thirty-four patients. *Ann Surg.*, **202**, 191–202.

[3] Demertzis, S., Ringe, B., Gulba, D., Rosenthal, H. and Pichlmayr, R. (1994). Treatment of portal vein thrombosis by thrombectomy and regional thrombolysis. *Surgery*, **115**, 389–393.

[4] Bezzi, M., Broglia, L., Lemos, A. A. and Rossi, P. (1995). Transjugular intrahepatic portosystemic shunt in portal vein thrombosis: role of the right gastric vein with anomalous insertion. *Cardiovasc. Intervent Radiol.*, **18**, 102–105.

[5] Tzakis, A., Todo, S., Stieber, A. and Starzl, T. E. (1989). Venous jump grafts for liver transplantation in patients with portal vein thrombosis. *Transplantation*, **48**, 530–531.

[6] Pinna, A. D., Lim, J. W., Sugitani, A. D., Starzl, T. E. and Fung, J. J. (1996). "Pants" vein jump graft for portal vein and superior mesenteric vein thrombosis in transplantation of the liver. *J Am Coll. Surg.*, **183**, 527–528.

[7] Benmark, S. and Hafstrom, Lo. (1969). The natural history of primary and secondary malignant tumours of the liver. I: The prognosis for patients with hepatic metastases from colonic and rectal carcinoma by laparotomy, *Cancer*, **23**, 198–202.

[8] Hodgson, H. J. F. (1994). Primary hepatocellular carcinoma. In: Blumgart LH., Ed. Surgery of the liver and biliary tract. London: Churchill Livingstone, pp. 1341–1347.

[9] Stevens, W. R., Johnson, C. D., Stephens, D. H. and Nagorney, D. M. (1995). Fibrolamellar hepatocellular carcinoma: stage at presentation and results of aggressive surgical management. *AJR. Am J Roentgenol.*, **164**, 1153–1158.

[10] Starzl, T. E., Iwatsuki, S., Shaw-BW, J., Nalesnik, M. A., Farhi, D. C. and Van, T. D. (1986). Treatment of fibrolamellar hepatoma with partial or total hepatectomy and transplantation of the liver. *Surg Gynecol. Obstet.*, **162**, 145–148.

[11] Erhard, J., Lange, R., Giebler, R., Rauen, U., de, G. H. and Eigler, F. W. (1995). Arterialization of the portal vein in orthotopic and auxiliary liver transplantation. A report of three cases. *Transplantation*, **60**, 877–879.

[12] Morimoto, T., Terasaki, M., Higashiyama, H., *et al.* (1992). Clinical application of arterialisation of portal vein in living related donor partial liver transplantation. *Transplant Int.*, **5**, 15.

[13] Tamura, M., Nakajima, Y., Kimura, J., *et al.* (1994). Experimental study on temporary portal vein arterialisation in porcine liver transplantation. *Transplant Proc.*, **26**, 2199.

[14] Mimura, H., Kim, H., Ochiari, Y., *et al.* (1988). Radical block resection of hepatoduodenal ligament for carcinoma of the bile duct with double catheter by pass for portal circulation. *Surg Gynecol. Obstet.*, **167**, 527.

[15] Maillard, J. N., Benhamou, J. P. and Rueff, B. (1970). Arterialization of the liver with portacaval shunt in the treatment of portal hypertension due to intrahepatic block. *Surgery*, **67**, 883–890.

